# Synthesis and Characterization of Novel Integral Asymmetric Monophasic Cellulose–Acetate/Silica/Titania and Cellulose–Acetate/Titania Membranes

**DOI:** 10.3390/membranes10090195

**Published:** 2020-08-20

**Authors:** Inês Peixoto, Mónica Faria, M. Clara Gonçalves

**Affiliations:** 1 Department of Biotechnology, Instituto Superior Técnico, Universidade de Lisboa, Av. Rovisco Pais, 1049-001 Lisbon, Portugal; inesparrinhapeixoto@gmail.com; 2 CeFEMA and Department of Chemical Engineering, Instituto Superior Técnico, Universidade de Lisboa, Av. Rovisco Pais, 1049-001 Lisbon, Portugal; 3 CQE and Department of Chemical Engineering, Instituto Superior Técnico, Universidade de Lisboa, Av. Rovisco Pais, 1049-001 Lisbon, Portugal; clara.goncalves@ist.utl.pt

**Keywords:** cellulose acetate/titania membranes, cellulose acetate/silica/titania membranes, monophasic hybrid membrane, nanocomposite membrane, ultrafiltration, sol-gel, phase inversion

## Abstract

Two series of novel integral asymmetric monophasic hybrid membranes, cellulose acetate/silica/titania (CA/SiO_2_/TiO_2_—series 1) and cellulose acetate/titania (CA/TiO_2_—series 2), were developed by the coupling of sol-gel technology and a modified version of the phase inversion technique. SEM micrographs confirmed the integral asymmetric structure of all membranes. ATR-FTIR and ICP-OES results showed that, for the membranes in series 1, TiO_2_ is covalently bound to SiO_2,_ which, in turn, is covalently bound to CA, while for the membranes in series 2, TiO_2_ is directly and covalently bound to the CA matrix. Permeation experiments revealed that the permeation performance of the membranes in series 1 is unaffected by the introduction of TiO_2_. In contrast, the introduction of TiO_2_ in the series 2 membranes increased the hydraulic permeability by a factor of at least 2 when compared to the pristine CA membrane and that incremental additions of TiO_2_ further increased the *Lp*.

## 1. Introduction

**Figure 1:**
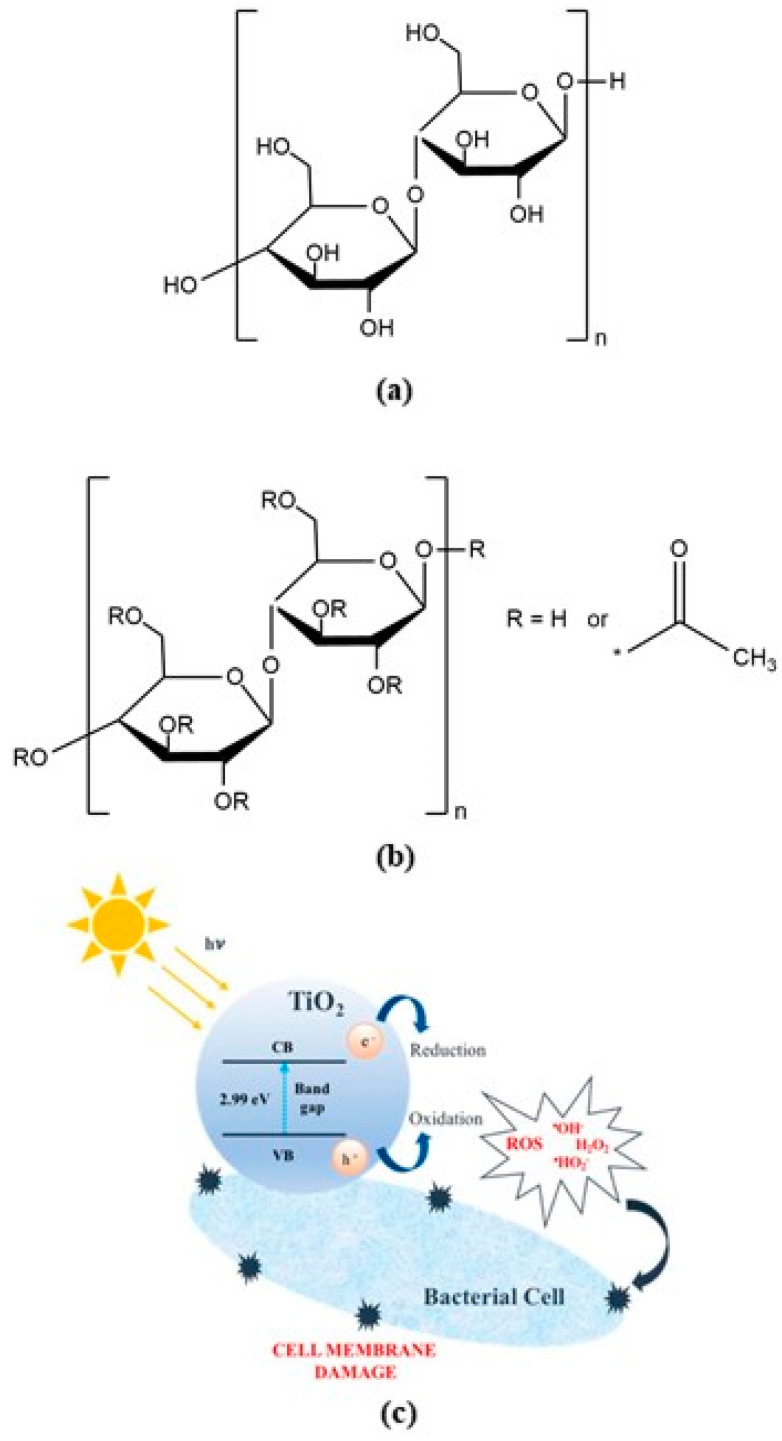
(**a**) Molecular structure of cellulose; (**b**) Molecular structure of cellulose acetate (CA); (**c**) Amorphous titania ROS production Illustration obtained with permission from Matos et al. [25].

Cellulose, a linear homopolymer composed of d–anhydroglucopyranose units linked by β–(1→4)–glycosidic bonds (Figure 1a), is one of the most abundant renewable organic materials produced in the biosphere, with approximately 5 × 10^11^ tons generated annually [1]. Cellulose’s most significant industrial ester, cellulose acetate (CA), is formed by the acetylation of cellulose, followed by acid-hydrolysis, to lower the acetyl degree (Figure 1b). Though this process modifies the original cellulose biostability, CA is generally recognized as a biodegradable [1,2], biocompatible and (re)absorbable thermoplastic polymer [3,4,5]. Properties such as excellent hydrophilicity, good resistance to chlorine and other oxidant compounds, non-toxicity, non-irritancy, great mechanical flexibility and reasonable heat-resistance, along with low footprint and cost make CA a pivotal raw material in a wide range of traditional industries [6]. Camera film (8 and 16 mm Kodak negative film), cigarette filters (Hoechst-Celanese, Eastman Chemicals, Rhodia Acetow, Daicel, and Mitsubishi Rayon), lacquers (Wurdack, Eastman), fibers (Celanese, Eastman, Viscocel, Mitsubishi Rayon), degradable plastics, toys (Lego bricks from 1949 to 1963), textiles (satins, brocades, and taffetas), magnetic tapes (IBM 726 tape drive in the IBM 701 computer) and separation-process membranes are among traditional CA products. The emergence of nanotechnology in recent years has brought valuable cellulose-based end-products, which span to engineering, pharmaceutical and medical fields. Technical and medicinal textiles, pharmaceutical drug coatings, microparticles for controlled drug release, sensors (immune-, chemical-, biochemical- and photosensors) and polymer membranes are some examples of engineered CA products in the market [6]. Regarding polymer membranes for industrial separation-processes, CA membranes have played an important role in drinking and wastewater treatment [7], gas separation [8], blood purification [9,10], ultra/nanofiltration and various other bio-separation processes [11,12], due to their high efficacy and cost effectiveness [13]. However, extreme operating conditions, such as high temperatures and/or low pH values, may promote the hydrolysis of the CA polymer, compromising the chemical, mechanical and thermal stability of the CA membranes. Furthermore, because cellulose is an organic polymer which can be digested by microorganisms, biofilms are known to grow at the active layer surface of CA-based membranes, a phenomenon known as biofouling, which may hinder the membrane productivity and its filtration efficiency, reducing the membrane lifetime and limiting its potential applications [14].

Membrane functionalization has been proven to be an efficient strategy towards biofouling prevention by promoting anti-adhesion surfaces [15] and/or controlling bacterial growth [16]. Most of the research being conducted in academic institutions and under industrial projects addresses two-phase systems, known as composites, where an inorganic additive such as zeolite [17], a transition metal complex [18], silver [19], silica [20], titania [21], fluorite [22], or a carbon nanotube [23] is incorporated into the polymer-based membrane casting solution. In these composites, repulsive forces between the polymer and the inorganic phases and/or different thermal expansion coefficients may compromise adhesion between the two composite phases, causing delamination or micro- or nano-particle loss during membrane operation.

The authors pioneered an alternative strategy in which monophasic (hybrid) CA/silica membranes were produced by in situ condensation between silanols (≡Si–OH^−^) from the SiO_2_ precursor and hydroxyl (–OH^−^) or acetate (–CH_3_COO^−^) groups from the CA polymer during casting solution homogenization [9,24]. Detailed characterization of the chemical composition of the monophasic hybrid CA/silica membranes revealed that the additive (SiO_2_) is covalently bound and homogeneously distributed throughout the polymer matrix [24].

Titania, either amorphous or crystalline, has been shown to possess an outstanding bactericidal performance, even without previous ultra-violet (UV) stimuli [25,26]. Although the mechanistic details of the TiO_2_ photocatalyzed reactions remain complex and are not completely understood [26,27], the role of TiO_2_ is widely recognized in the formation of reactive oxygen species (ROS) (hydroxyl, superoxide anion radicals, O_2_, O_2_^−^). When in close proximity to bacteria, these ROS’s damage bacterial cell membranes through lipid peroxidation, enhance membrane fluidity, and cause cell integrity disruption, eventually leading to cell death [26,27] (Figure 1c).

In this work, we report on a novel method for the development of integral asymmetric monophasic hybrid membranes by coupling a modified version of the phase inversion technique [28] and sol-gel methodology [29]. The sol-gel method enables the condensation between inorganic silica precursors (hydrolysed TEOS and TiPOT precursors, ≡Si–OH^−^, ≡Ti–OH^−^, respectively) and the organic phase, CA, during the preparation of the casting solution, promoting two distinct monophasic hybrid systems characterized by ≡Si–O–CA and ≡Ti–O–CA covalent bonding. In these systems, following the hydrolysis reaction shown in reactions 3a, Annex A, self-condensation may occur between Si–OH, Ti–OH or CA–CA monomers, creating small silica, titania or CA clusters spread throughout the CA–SiO_2_–TiO_2_ and CA–TiO_2_ monophasic hybrid membrane matrices. Hetero-condensation reactions (depicted in reaction 3b, Annex A) also occur and compete with the self-condensation reactions. Due to the very high number of hydrolyzed CA units over Si–OH and Ti–OH units, as well as the vigorous stirring during the preparation step of the casting solution, hetero-condensation reactions predominate over self-condensation reactions, leading to the synthesis of monophasic hybrid systems.The phase inversion technique is a well-known method for the preparation of integral asymmetric membranes, which, when coupled to the sol-gel method, promotes the synthesis of integral asymmetric monophasic hybrid CA/silica/titania (CA/SiO_2_/TiO_2_—series 1) and CA/titania (CA/TiO_2_—series 2) membranes. These membranes present only one phase, as opposed to composite membranes, where the inorganic component is added to the casting solution in the form of nano- or micro-particles.

The introduction of TiO_2_ in the casting solutions of the reference membranes, CA/SiO_2_ and CA, is expected to affect the permeation performance of the two new hybrid membranes, CA/SiO_2_/TiO_2_ and CA/TiO_2_. For the membranes in series 1, the CA organic content was kept constant and equal to 95 wt%. The inorganic content consisted of SiO_2_ and TiO_2_, and the TiO_2_ was increased by steadily replacing the silica content with titania. For the membranes in series 2, the inorganic phase was composed solely of titania, which was steadily increased from 0 to 5 wt%. A maximum total inorganic content of 5 wt% was chosen based on the previous works on permeation by the authors [9,24], which revealed enhanced permeation properties for monophasic hybrid integral asymmetric CA/silica membranes containing 5 wt% silica.

## 2. Materials and Methods

### 2.1. Materials

Series 1 (CA/SiO_2_/TiO_2_) and series 2 (CA/TiO_2_) monophasic hybrid asymmetric membranes were prepared with cellulose acetate (CA) (C_6_H_7_O_2_(OH)_3_, ≈ 30,000.00 g/mol, ≥97%), titanium(IV) isopropoxide (TiPOT) (C_12_H_28_O_4_Ti, 284.00 g/mol, 97%), tetraethyl orthosilicate (TEOS) (Si(OC_2_H_5_)_4_, 208.33 g/mol, reagent grade 98%), purchased from Sigma-Aldrich Quimica S.L. (Lisbon, Portugal), formamide (CH_3_NO, 45.02 g/mol, ≥99.5%), purchased from Carlo Erba Reagents (Barcelona, Spain), acetone (C_3_H_6_O, 58.08 g/mol, 99.7%) and nitric acid (HNO_3_, 63.01 g/mol, >60%), purchased from JMS (Lisbon, Portugal).

Membrane drying was performed with isopropanol (25% V/V, 50% V/V, 75% V/V, 100% V/V) (anhydrous, 99.5%) from Sigma-Aldrich Aldrich Quimica S.L. (Lisbon, Portugal) and n–hexane (25% V/V, 50% V/V, 75% V/V, 100% V/V) (98%) from Sigma-Aldrich Aldrich Quimica S.L. (Lisbon, Portugal).

The solutes used in permeation experiments were 1, 3, 4, 6, 10, 20, 35, and 55 kDa polyethylene glycol (PEG) from Sigma-Aldrich Aldrich Quimica S.L. (Lisbon, Portugal) and 70 kDa Dextran from Sigma-Aldrich Aldrich Quimica S.L. (Lisbon, Portugal).

Bi-distilled water (H_2_Od) (conductivity 0–2 µS/cm^3^, pH 5.8–6.5) and pure deionized (DI) water were obtained at Laboratório de Análise de Águas, IST, Lisbon, Portugal.

All chemicals used in the synthesis, drying and characterization of the hybrid membranes were used without further purification.

### 2.2. Membrane Synthesis

Membranes were synthesized by a modified version of the phase inversion technique [28] coupled with sol-gel methodology [29]. The acid catalyzed hydrolysis and condensation sol-gel reactions took place during the preparation of the casting solution (24 h period). During this period, the polymerization between the hydrolyzed inorganic species containing ≡Si–OH^−^ and ≡Ti–OH^−^ groups and the CA polymer containing –OH– and –CH_3_COO– groups occurs, generating monophasic hybrid matrices containing ≡Si–O–CA and ≡Ti–O–CA groups. The complete sol-gel hydrolysis and condensation reactions are presented in detail in Appendix A.

Two distinct groups of membranes were produced: series 1—CA/SiO_2_/TiO_2_ membranes—and series 2—CA/TiO_2_ membranes. For each membrane composition, one batch was produced, resulting in ten flat sheet membranes with dimensions of approximately 20 cm (width) × 30 cm (height) each. The membranes’ acronyms and casting solution compositions are shown in Table 1. The acronyms reflect the membranes’ composition, i.e., the mass percentage of CA, SiO_2_, and TiO_2_ in the synthesized membranes. Appendix B shows a detailed description of these calculations. After complete sol-gel hydrolysis and condensation, each TEOS molecule originates one silica unit, and each TiPOT molecule originates one titania unit. Formamide and acetone (solvents) and water (sol-gel reactant) were discarded in the calculations.

#### 2.2.1. Series 1: CA/SiO_2_/TiO_2_ Membranes

For the preparation of the membranes in series 1, five different casting solutions were prepared. In this membrane set, the organic content was kept constant (95 wt% CA) with an inorganic part consisting of SiO_2_ and TiO_2_, while SiO_2_ was steadily replaced by TiO_2_, as shown in Table 1, Appendix B. In series 1, the CA/SiO_2_ (95/5) membrane was selected as the reference membrane.

One-pot sol-gel (hybrid) reactions were promoted during the casting solution homogenization. The acid catalyzed hydrolysis/condensation of TEOS and TiPOT (the SiO_2_ and TiO_2_ alkoxide precursors, respectively) was promoted in situ by adding TEOS, TiPOT, DI, and nitric acid to the CA polymer and solvent (formamide and acetone) mixture in the reaction vessel. TEOS was added, drop-by-drop, at the beginning of the casting solution homogenization, while TiPOT was added, drop-by-drop, 8 h later, due to its faster hydrolysis rate. To help with the TiPOT solubilization in the casting solution, TiPOT was pre-solubilized in a solution of nitric acid, formamide and acetone.

After 24 h of homogenization, the hybrid casting solutions were poured onto a glass plate, at room temperature, with a 250 μm casting knife. After a solvent evaporation time of 30 s, the glass plates were quenched into a gelation bath (ice-cold H_2_Od). After being in the coagulation bath for 2 h, the membranes were detached from the glass plate and stored in H_2_Od at 4 °C.

#### 2.2.2. Series 2: CA/TiO_2_ Membranes

For the preparation of the membranes in series 2, five different casting solutions were prepared with CA and an increasing inorganic content of TiO_2_ (up to 5 wt%), as shown in Table 1, Appendix B. In series 2, CA was chosen as the reference membrane. Acid catalyzed hydrolysis/condensation of TiPOT, the TiO_2_ precursor, was promoted in situ by adding DI, TiPOT, drop-by-drop, and nitric acid to the CA/formamide/acetone mixture in the reaction vessel. The membranes were casted in the same conditions as described for the membranes in series 1.

### 2.3. Membrane Drying

The membranes in series 1 and 2 were dried by the solvent-exchange method [30]. Briefly, the membranes were submerged in several different solutions for a period of 24 h at room temperature, followed by the final solvent (100% n–hexane) evaporation in a desiccator. A detailed sequence of the solvent system used and immersion time is given in Appendix C.

### 2.4. Membrane Characterization

#### 2.4.1. FEG-SEM: Surface Morphology and Cross-Section Structure

The membrane surface morphology and cross-section structure were analyzed by Field Emission Gun–Scanning Electron Microscopy (FEG-SEM) (JEOL 7001F JOEAL). SEM images of the top and bottom layers were taken at 5000× magnification and the cross sections were taken with a magnification between 550 and 650×.

Prior to being imaged, the membranes were cut (1 × 1 cm) and dried according to the process described in Section 2.3. The dried membranes were fractured in liquid nitrogen, mounted on a stub and sputter-coated with gold. The membranes’ total thickness and skin layer thickness were measured from FEG-SEM cross-section images with ImageJ software. For each membrane, five randomly selected zones from the entire cross-section images were measured and the mean thickness and standard deviation were calculated, as shown in Appendix D. Light elements energy-dispersive X-Ray spectroscopy (EDS) was used to evaluate the presence of Ti.

#### 2.4.2. ICP-OES: Quantification of Titanium

Inductively Coupled Plasma Optical Emission Spectrometry (ICP-OES) (ICP-OES Optima 2000 from PerkinElmer, USA) was used to quantify the presence of titanium in the series 1 and series 2 membranes. Prior to being analyzed, four different wet samples of each membrane composition with a surface area of 16 cm^2^, from at least two different membranes from the same batch, were dried at room temperature until constant weight was achieved. Then, they were placed in an Heraeus oven at 550 °C for 30 min to discard volatile components and organic matter and break intra- and intermolecular bonds. After cooling to room temperature, the ashes were solubilized in acidified water (pH ≤ 2). The solution was then introduced in the ICP-OES equipment and plasma-atomized. After excitation, Ti decayed to ground state and the emitted photons (*hv*) allowed for an accurate and precise Ti quantification (<3 wt%). This assay was done in triplicate, and the mean mass and standard deviation were determined.

The predicted theoretical titanium mass, *m_Ti(theoretical)_*, was calculated assuming homogenous distribution of Ti throughout all 10 membranes of each batch. The *m_Ti(theoretical)_* was defined by

(1) $$m_{Ti\left(theoretical\right)}=\frac{w_{m}\times n_{TiPOT\times MW_{Ti}}}{M_{T}}$$

and the calculations were based on a 16 cm^2^ surface area membrane sample. $m_{Ti\left(theoretical\right)}$ is the predicted theoretical mass of titanium in the membrane sample; $w_{m}$ is the membrane sample weight; $n_{TIPOT}$ is the number of TiPOT moles introduced in the membrane casting solution (each TiPOT molecule originates one TiO_2_ unit); $MW_{Ti}$ is the molecular weight of titanium; and $M_{T}$ is the mass of all 10 membranes obtained in the batch. $M_{T}$ is calculated taking into account the mass of CA, TiO_2_ (obtained assuming complete hydrolysis and condensation of TiPOT) and SiO_2_ (obtained assuming complete hydrolysis and condensation of TEOS) per batch ($M_{T}=m_{CA}+m_{TiO2}+m_{SiO2}$).

The error propagation through the m_Ti_ calculation was determined. The uncertainties are: (1) 0.01 g for the CM-600 CBJ scale (Barcelona, Spain) used to weigh all chemicals and (2) 0.0001 g for the Sartorious BA120s scale (Göttingen, Deutschland) used to weigh the membrane samples. The sum of absolute errors (*δ*x) of the measured variables (x) was considered for additions or subtractions. In multiplications or quotients, the sum of the relative errors $\left(\frac{\delta x}{\left|x\right|}\right)$ was used, assuming errors < 10% [31,32,33].

#### 2.4.3. ICP-OES: Quantification of Leached Titanium

**Figure 2:**
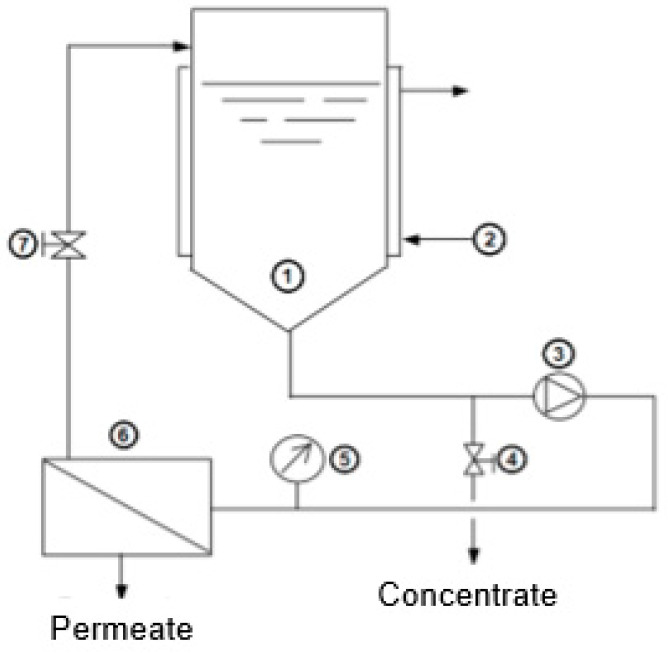
Schematic representation of the Celfa-P28 UF experimental set-up: (1) feed tank; (2) heating/cooling jacket; (3) pump; (4) valve of concentrate collector; (5) manometer; (6) permeate cell; (7) pressure retention valve.

To study the possibility of TiO_2_ leaching from the synthesized membranes, a pure DI water permeation assay was conducted with the membranes containing the highest TiO_2_ content in both series 1 and series 2 (described in Section 2.5, Figure 2). The assay was performed in triplicate for each composition (samples from different membranes with the same composition were analyzed). Mean Ti concentration and standard deviation were calculated.

The permeation experiment consisted in recirculating 500 mL of pure deionized (DI) water at a feed flow rate (*Q_f_*) of 0.6 L/min and a transmembrane pressure (TMP) of 2 bar in the Celfa-P28 cross-flow filtration system described in Section 2.5, Permeation Properties. Samples of the initial feed solution (pure DI water) and samples from the permeate and concentrate collected after 3 h of filtration were analyzed by ICP-OES to quantify Ti.

#### 2.4.4. ATR-FTIR: Chemical Bonding

The active layer surface of all membranes, both from series 1 and series 2, was analyzed by Fourier transform infrared spectroscopy (FTIR) in attenuated total reflection (ATR) mode. Prior to characterization, random samples with a surface area of approximately 1 cm^2^ were air-dried at room temperature. FTIR spectra of the samples (five per membrane composition), with the active layer facing upwards, were obtained with a Nicolet Magna IR System 5700 spectrometer (Nicolet Instrument Corp., Madison, WI, USA) using a Golden Gate MKII ATR accessory with a Ge crystal (Graseby Specac, Smyrna; sampling depth: 0.2–1.1 μm at 4000–400 cm^−1^). Each spectrum was obtained by averaging 256 scans with a resolution of 4 cm^−1^. The spectra were transformed to log_10_(1/R) using OMNIC software and are presented without baseline or smooth corrections.

The complete ATR-FTIR spectra (825–3750 cm^−^^1^) were recorded to identify the types of bonds present in all membranes. CA/SiO_2_ 95:5, in series 1, and CA 100, in series 2, were taken as reference.

In order to compare the chemical structure of the hybrid membranes from series 1 and series 2, a more detailed analysis was performed in regions 1690–1800 cm^−1^ and 950–1175 cm^−1^ of the carbonyl absorbance band where Ti–O–C peaks are expected [34,35,36,37].

The 1000–1110 cm^−1^ ATR-FTIR bands from series 1 and series 2 were decomposed by Gaussian curve-fitting (Levenberg Marquardt algorithm, allowing variation in width, height, and position of the bands) after a baseline correction (subtraction of a straight line between two extreme wavenumbers of the region). The number and starting position of the bands used in the fitting were obtained from the smoothed (Savitzky–Golay algorithm) second-derivative spectrum of the region. For each membrane, two individual peaks were found, and a non-linear least-squares fitting procedure was performed to obtain 100% Gaussian shaped peaks. The ATR-FTIR spectra data was analyzed using Origin 8 pro software (https://www.originlab.com/).

### 2.5. Permeation Properties

#### 2.5.1. Hydraulic Permeability

Figure 2 shows a schematic representation of the experimental ultrafiltration (UF) crossflow set-up used to determine the pure water hydraulic permeability (*Lp*) of the membranes from series 1 and series 2.

The permeation cell of Celfa-P28 consists of two detachable parts separated by a porous plate to accommodate a membrane with a total surface area of 25 cm^2^. Prior to each experiment, the membrane was compacted for 3 h with deionized water at a transmembrane pressure (TMP) 20% higher than the maximum operating pressure.

The membranes were characterized as to pure water permeation fluxes, hydraulic permeability, and separation performance in terms of apparent rejection coefficients for several PEG and Dextran solutions.

The hydraulic permeability coefficient (*Lp*) is defined by

(2) $$Lp=\frac{Jpw}{TMP}$$

where Jpw is the pure water permeation flux and *TMP*.

The pure water fluxes in the series 1 and series 2 membranes were measured using DI water at room temperature under two different experimental conditions: (i) feed flow rate (*Q_f_*) of 0.6 L/min and TMPs of 0.5, 1.9, 1.5, 2.0, 2.5, and 3.0 bar; (ii) *Q_f_* of 1.3 L/min and TMPs of 1.0, 1.5, 2.0, 2.5, 3.0, and 3.5 bar. For each *Q_f_*/TMP condition, *Jpw* was measured in triplicate.

#### 2.5.2. Molecular Weight Cut-Off

The membrane molecular weight cut-off (MWCO), which is defined as the molecular weight of a macromolecule whose rejection by the membrane is higher than 90%, was experimentally determined through ultrafiltration (UF) crossflow, whose set-up is described in Figure 2. The MWCO values of two membranes from series 1 (95/5/0, and 95/2/3 CA/SiO_2_/TiO_2_) and two from series 2 (100, and 95/5 CA/TiO_2_) were determined by the apparent solute rejection coefficients (*f*) defined by

(3) $$f=\frac{C_{b}-C_{p}}{C_{b}}$$

where *C_b_* and *C_p_* are the solute concentrations in the bulk feed solution and permeate solution, respectively. Permeation experiments were performed to calculate the apparent rejection coefficients for PEG 1000, 3000, 4000, 6000, 10,000, 20,000, 35,000, 55,000, and Dextran 70,000. All experiments were conducted at room temperature, with single solute solutions, feed concentration of 600 ppm, feed flow rate of 93 L/h, and transmembrane pressure of 1 bar. The concentrations of the PEG and Dextran solutions in the feed and in the permeate were determined in terms of total organic carbon content by a Total Organic Carbon Analyzer (Dohrmann Total Organic Carbon Analyzer Model DC-85A, Tokyo, Japan). The MWCO value was determined by a linear regression of the curve *log*(*f/1* − *f*) vs. the molecular weights of the organic solutes and its intersection with the 90% rejection line, which corresponds to a *log*(*f/1* − *f*) of 0.954.

## 3. Results and Discussion

### 3.1. Membrane Structure and Surface Morphology

**Figure 3:**
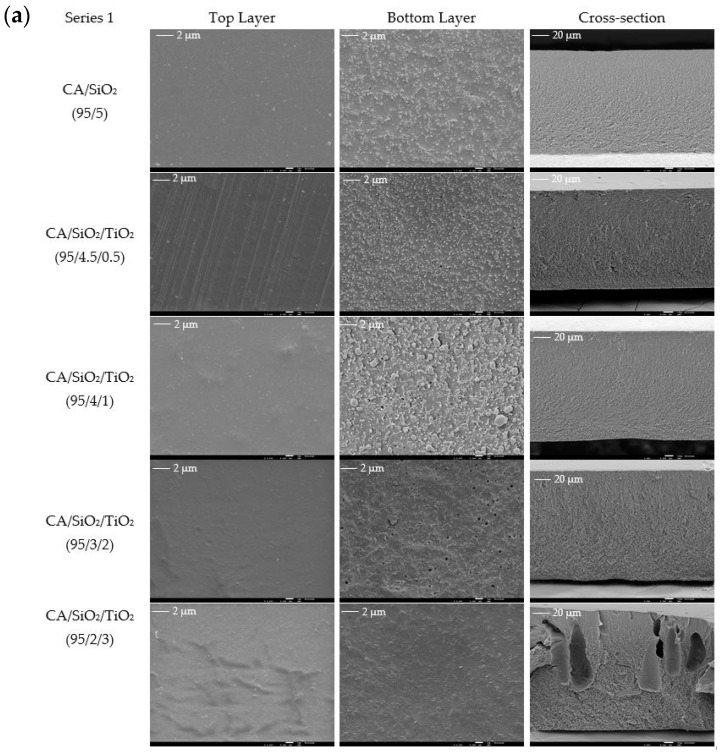

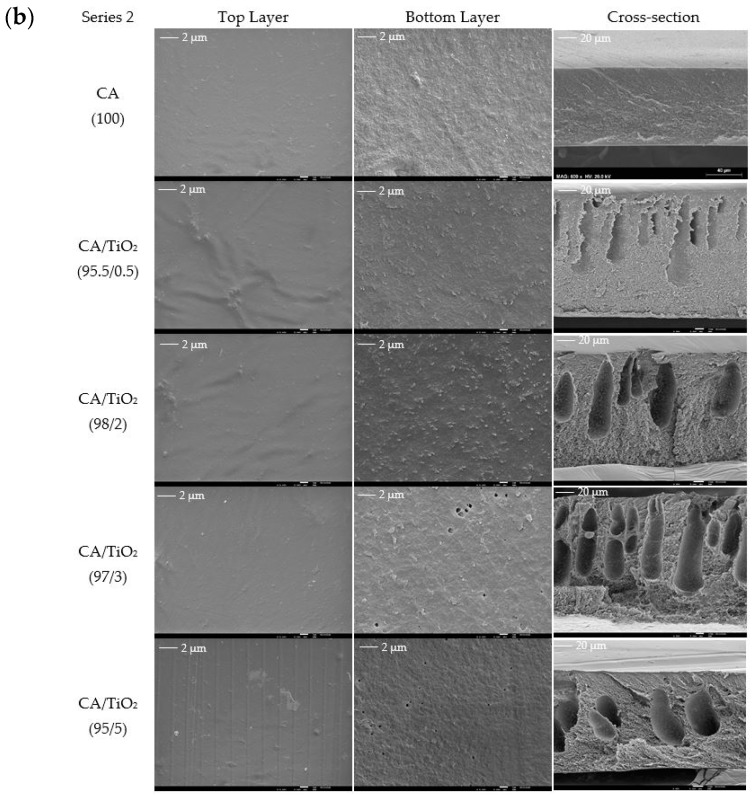
Field emission gun (FEG)-SEM images of the top surface (magnification 5000×), bottom surface (magnification between 550× and 650×) and cross-sections (magnification 5000×) of: (**a**) the membranes in series 1, and (**b**) the membranes in series 2.

The top and bottom surfaces and cross-section morphologies of the membranes from series 1 and series 2 analyzed by SEM and micrographs are shown in Figure 3a,b, respectively. The SEM images obtained from the top surfaces clearly show smooth, non-porous and dense membrane morphologies in both series 1 and series 2. In contrast, the membranes’ bottom surfaces appear to have a more uneven and porous structure. The cross-section SEM images clearly identify the membranes’ integral asymmetric nature in both series, which is characterized by a thin skin dense layer on top (active layer), outlining a thicker and porous substructure (porous layer). As a first conclusion, the introduction of TiO_2_ on the membranes’ composition did not affect the membranes’ asymmetric character in neither series 1 nor series 2. Nevertheless, finger-like macro-voids appear in the series 1 membranes (Figure 3a) with higher TiO_2_ content (CA/SiO_2_/TiO_2_, 95/2/3) and in all series 2 membranes (Figure 3b). According to Smolders et al. [38], macro-void formation occurs under rapid precipitation conditions and/or a decrease in the viscosity of the casting solution. In the present work, adding higher contents of TiPOT decreased the viscosity of the casting solutions in both series 1 and series 2, which may explain the macro-void configuration.

**Figure 4:**
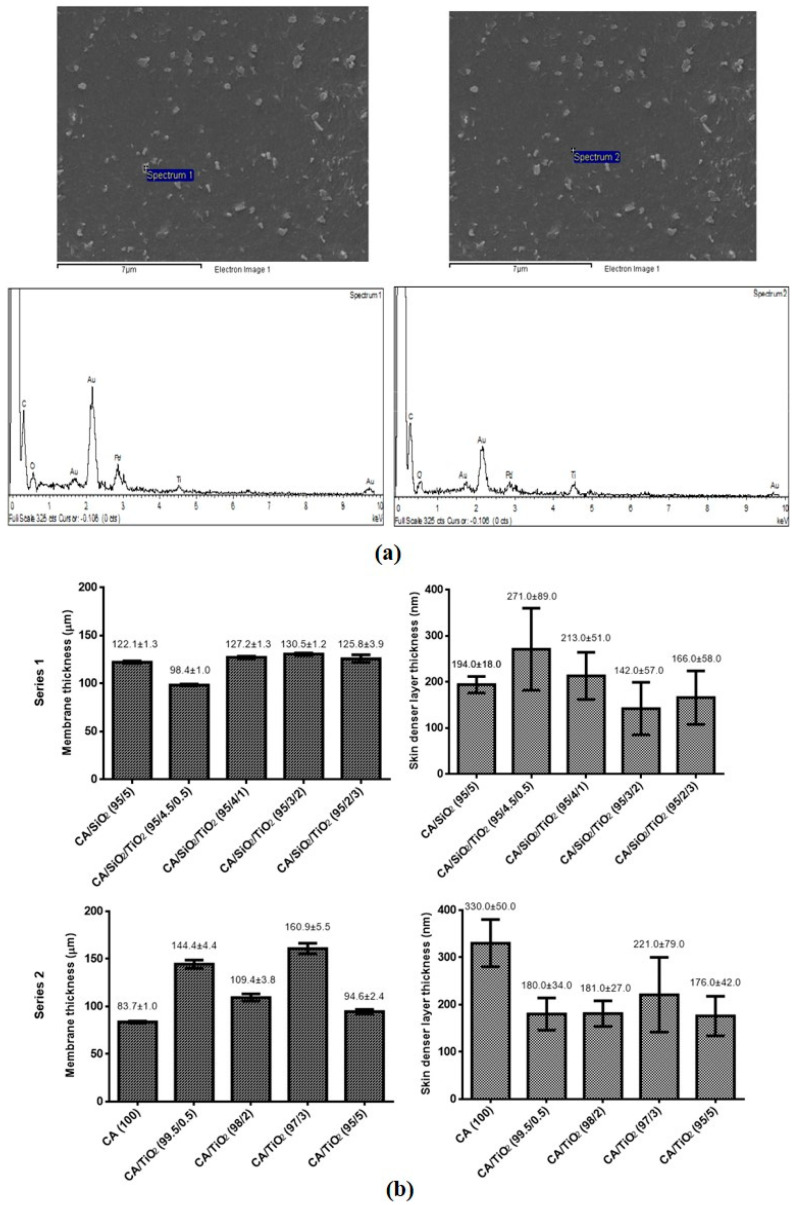
(**a**) Bottom SEM images of the CA/TiO_2_ (98/2) membrane analyzed by EDS. Spectrum 1 EDS analysis was performed in a surface deposit and spectrum 2 EDS analysis was performed in the bottom flat surface of the membrane. (**b**) Membrane thickness (µm) and skin dense layer (nm) thickness for the membranes in series 1 and series 2. The total membrane thickness and dense layer thickness were measured from the SEM images of the series 1 and series 2 membranes’ cross sections using ImageJ software (procedure illustrated in Appendix D).

Membranes 95/4.5/0.5 and 95/4/1 (from series 1, CA/SiO_2_/TiO_2_) and 99.5/0.5 and 98/2 (from series 2, CA/TiO_2_) showed distinct features on the bottom surfaces, which were further investigated by EDS. Figure 4a shows SEM images and the EDS spectrum of the 98/2 membrane (series 2, CA/TiO_2_), revealing identical Ti peak intensities in the deposit spectra (spectrum 1) and the bottom flat surface (spectrum 2, reference membrane). Ti peak intensity is even lower in spectrum 1 (deposit) than in spectrum 2 (the membrane’s bottom surface), ruling out the hypothesis of TiO_2_ segregation.

The SEM images were used to determine the total thickness and the dense layer thickness of the membranes from series 1 and series 2. The procedure adopted for measuring the dense layer thickness of the CA/SiO_2_/TiO_2_ (95/4/1) membrane from series 1 is shown in Appendix D. For the membranes in series 1, the total thickness varies between 98 and 130 µm and the dense layer thickness varies between 142 and 271 nm. The introduction of TiO_2_ into the membrane casting solutions and its incremental increase do not seem to have a direct influence on the total or dense layer thickness of the CA/SiO_2_/TiO_2_ membranes. For the membranes in series 2, the total thickness varies between 84 and 161 µm and the dense layer thickness varies between 176 and 333 nm. The introduction of TiO_2_ into the membrane casting solutions and its incremental increase do not seem to have direct influence on the total thickness of the CA/TiO_2_ membranes. However, there is a significant reduction in the dense layer thickness of the membranes containing TiO_2_ when compared to the pristine CA membrane. The skin layer thickness of the CA membrane (330 ± 50 nm) is approximately 1.7 times larger than the average value of the dense layer thickness (189 nm) of the membranes containing TiO_2_ in series 2.

In integral asymmetric membranes, the dense layer thickness determines the resistance to mass transport, with the filtration efficiency being inversely proportional to the dense layer thickness [13]. All series 2 membranes containing TiO_2_ and two membranes in series 1, CA/SiO_2_/TiO_2_ (95/3/2) and CA/SiO_2_/TiO_2_ (95/2/3), exhibited lower skin dense layer thickness than the reference (CA/SiO_2_) membrane. Therefore, these membranes are expected to impose less mass transport resistance to solutions and higher permeation capacities are envisaged, which is the reason why they were selected for determining the MWCO.

### 3.2. Titanium Quantification

#### 3.2.1. Titanium Incorporated in the Membranes

The titanium (Ti) content in the series 1 and series 2 membranes was evaluated by ICP-OES and the results of the experimental and theoretical Ti content are shown in Table 2. Results show that the experimental results and theoretical estimations coincide and are within the experimental error, which leads to the conclusion that the entire amount of Ti introduced in the casting solution, through the addition of TiPOT, is present in the monophasic hybrid membranes. Furthermore, the variation between different samples from the same batch is low, suggesting a homogeneous Ti distribution throughout the entire membrane.

#### 3.2.2. Titanium Leached from the Membranes

The quantification of Ti leached from the membranes was determined in a permeation experiment, through ICP-OES analysis of the H_2_Od collected from the permeate and concentrate solutions. Table A3 in Appendix E shows the values of Ti found in each experiment. The initial and final permeate and concentrate values are the same in all solutions, <0.010 ± 0.000 mg/L, and are within the experimental values for all membranes from series 1 and series 2. These results mean that, during the 3 h filtration, the membranes did not release any titanium, which indicates a strong bonding between titania and the CA membrane matrix. Since the membranes with higher TiO_2_ content from series 1 and series 2 did not leach Ti, it is unlikely that the remaining membranes with lower TiO_2_ content will.

### 3.3. Chemical Composition and Hybrid Bonding

#### 3.3.1. Series 1 Membranes

**Figure 5:**
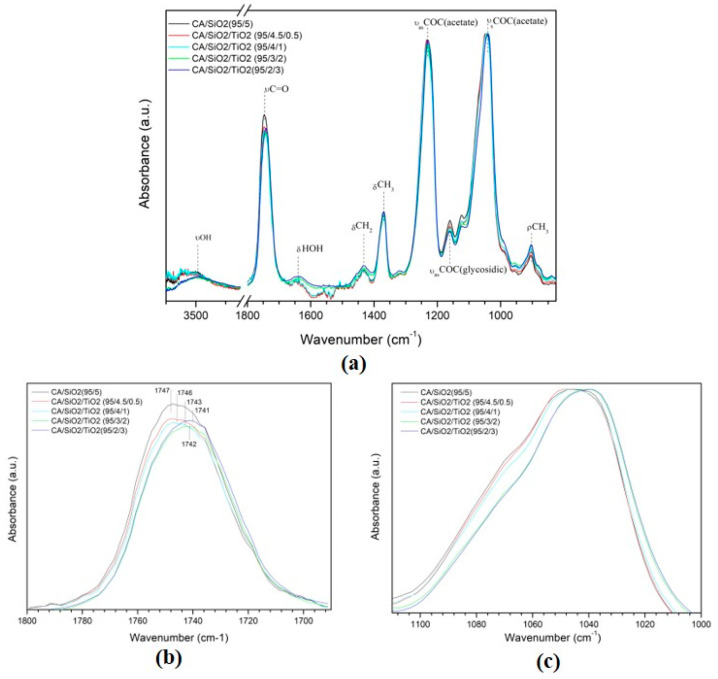
Attenuated total reflection (ATR) spectra of the active layer of CA/SiO_2_/TiO_2_ membranes normalized to the C=O stretching band: (**a**) complete spectrum; (**b**) enhanced 1690–1800 cm^−1^ region, and (**c**) enhanced 1000–1110 cm^−1^ region.

Figure 5 shows ATR-FTIR spectra for the series 1 membranes. In Figure 5a, the wide-range ATR-FTIR spectra (825–3750 cm^−1^), normalized to the C=O stretching band, is presented and, in Table 3, peak assignments are given. The 95/5 spectrum confirms the previous work from the authors [24]. The most intense peaks (equal intensity for all membranes in series 1) are the CA peaks found at 1045 and 1232 cm^−1^, which are assigned to acetate groups (symmetric (ν_s_COC) and antisymmetric (ν_as_COC) modes, respectively [39,40]). The band centered at 1369 cm^−1^ is assigned to δ_s_CH_3_, and the band centered at 1741 cm^−1^ is assigned to the strong carbonyl stretching mode (νC=O) [41,42]. The broad band located at 3000–3700 cm^−1^ (assigned to the O–H stretching mode, νOH) [39] contains contributions from water (H–O–H), hydrolyzed silica/titania precursors (≡Si–OH^−^, ≡Ti–OH^−^), and non-esterified cellulose polymer –OH groups. The three weaker peaks found at 1065, 1122, and 1161 cm^−1^ are assigned to C–O–C stretching modes (from glycosidic bonds and monosaccharide units, ν_as_COC) [43]. Since the esterification degree of the cellulose acetate is 40%, the glycosidic oxygens can establish intramolecular hydrogen bonds within the CA hydroxyl groups. Two important bands of CA/SiO_2_ hybrid membranes are the ν(Si–O–Si) and ν(Si–O–C), observed at 1065 and 1118 cm^−1^, respectively [24]. Lower intensity peaks are observed at 1433 and 1637 cm^−1^ (assigned to δCH_2_ and δHOH, respectively) [41,42]. The weak band centered at 903 cm^−1^ confirms the presence of acetate methyl groups [39]. To highlight specific characteristics within series 1, deconvolution studies in the 1690–1800 cm^−1^ and 1000–1100 cm^−1^ regions were performed.

Figure 5b documents the carbonyl region with stretching modes located between 1690 cm^−1^ and 1800 cm^−1^. Table 4 presents the carbonyl peak areas (A) and wavenumbers corresponding to this region. Incremental additions of TiO_2_ shift the carbonyl peak to lower wavenumbers (blue shift) and, at the same time, reduce the carbonyl peak area. This is a clear indication of Ti–CA bonding formation by nucleophilic substitution of the carbonyl (C=O) rather than by the hydroxide (OH) group (Appendix A). Furthermore, the νC=O shift indicates bond length increase and, consequently, bond weakening.

**Figure 6:**
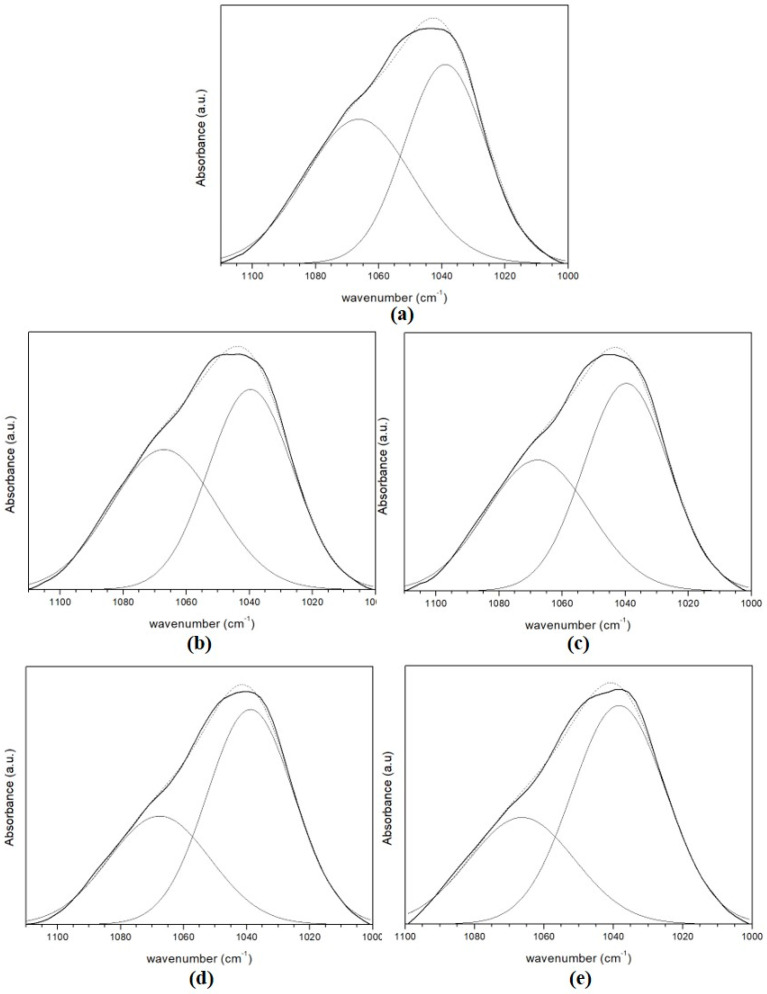
ATR-FTIR spectra of the hybrid membranes from series 1 in the 1000–1110 cm^−1^ region. Curve-fitting decomposition of the bands located between 1000 and 1110 cm^−1^ for: (**a**) CA/SiO_2_(95/5); (**b**) CA/SiO_2_/TiO_2_ (95/4.5/0.5); (**c**) CA/SiO_2_/TiO_2_ (95/4/1); (**d**) CA/SiO_2_/TiO_2_ 95/3/2, and (**e**) CA/SiO_2_/TiO_2_ 95/2/3 membranes. Bold black line—experimental results; dashed line—simulated results.

Figure 5c shows the region of the ATR-FTIR spectra between 1000 and 1110 cm^−1^ for the membranes in series 1. Careful analysis of these curves shows that there are contributions from two main peaks centered at approximately 1040 and 1067 cm^−1^. Figure 6 shows the curve-fitting decomposition of the region between 1000 and 1110 cm^−1^ for the CA/SiO_2_/TiO_2_ membranes, and Table 5 shows the values of the wavelength and relative peak areas. The peak centered at 1039 cm^−1^ corresponds to the symmetric COC vibrations of the acetate group (ν_s_COC (acetate)) [39,40], and the peak centered at 1067 cm^−1^ corresponds to ν_a_SiOSi [41,42,44,45,46]. This indicates the formation of a Si–O–Si network, probably in nano/micro-clusters dispersed throughout the polymer matrix [24].

#### 3.3.2. Series 2 Membranes

**Figure 7:**
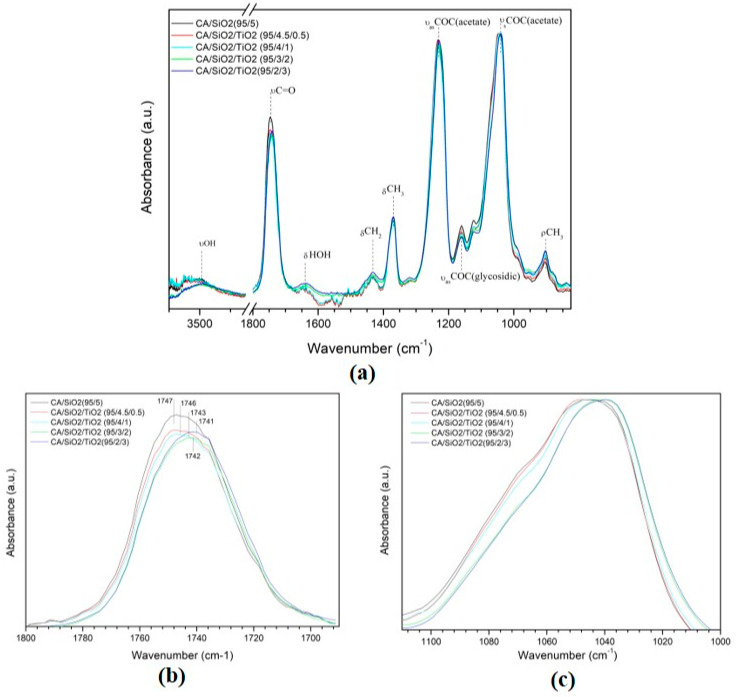
ATR-FTIR spectra of the reference pristine CA membrane and the hybrid CA/TiO_2_ membranes from series 2 (normalized to the C=O stretching band), (**a**) wide spectrum (4000–800 cm^−1^); (**b**) enhanced 1690–1800 cm^−1^ region and (**c**) enhanced 1000 and 1110 cm^−1^ region.

Figure 7 shows the ATR-FTIR spectra for series 2 membranes. In Figure 7a, the wide ATR-FTIR spectra between 825 and 3750 cm^−1^ of the reference membrane (composed solely of CA) and the hybrid membranes containing between 0.5 and 3 wt% TiO_2_, normalized to the C=O stretching band, is evidenced. The complete assignment of the spectra is presented in Table 6. The same intense bands corresponding to CA which were seen for the membranes in series 1 are also present in all series 2 membranes. To highlight the differences between the pristine CA membrane and the hybrid CA/TiO_2_ membranes, the enhanced region of the carbonyl stretching mode (νC=O) between 1680 and 1800 cm^−1^ is presented in Figure 7b. Table 7 identifies the peak area and wavenumbers of the carbonyl group for series 2 membranes. Results clearly show an increase in the peak area corresponding to the carbonyl group, along with a shift from 1739 cm^−1^ for the reference CA membrane to 1744 cm^−1^ for the CA/TiO_2_ 99.5:0.5 membrane, 1745 cm^−1^ for the CA/TiO_2_ 98:2 membrane, and 1749 cm^−1^ for the CA/TiO_2_ 97:3 and CA/TiO_2_ 95:5 membranes. The CA content in all membranes is equal, so the fact that the peak assigned to νC=O is increasing indicates that carbonyl groups are not being removed from the CA molecule when TiO_2_ is introduced and, therefore, TiO_2_ is bonding to CA by nucleophilic substitution of the hydroxyl (OH) group rather than the carbonyl (C=O) group of CA (Appendix A).

Figure 7c shows the ATR-FTIR absorption spectra of the CA membrane and the CA/TiO_2_ membranes in the region located between 1000 and 1110 cm^−1^. It is evident that the center of the peak shifts from 1043 cm^−1^ for the pristine CA membrane to a lower wavenumber of 1037 cm^−1^ for the membranes containing TiO_2_ and that the total area of the band belonging to the pristine CA membrane is larger than the bands corresponding to the hybrid CA/TiO_2_ membranes. For the pure CA membrane, the peak centered at 1043 cm^−1^ has the contribution of two groups: the symmetric COC vibrations of the acetate group (ν_s_COC (acetate)), and the antisymmetric COC vibrations of the glycosidic group (ν_as_COC (glycosidic)). Both these groups are present in the hybrid CA/TiO_2_ membranes and, therefore, the shift to higher wavenumbers can be explained by the contribution of the band assigned to the stretching vibration of the Ti–O–C group (νTiOC), which absorbs at higher wavenumbers located between 1039 and 1045 cm^−1^ [35,36]. The presence of Ti–O–C bonds gives evidence that covalent bonds between the inorganic (TiO_2_) and organic (CA) components of the hybrid CA/TiO_2_ membranes were established in series 2.

### 3.4. Permeation Properties

#### Hydraulic Permeability and MWCO

The hydraulic permeability coefficient (*Lp*) reveals the pure water permeation capacity of a membrane. For the *Lp* determination, pure water permeate fluxes (*Jpw*) were measured at different pressures and flow rates. For each membrane in both series, the linear regression of the *Jpw* in function of the transmembrane pressures (∆*P*) applied was obtained. The slope of the linear regression equation gives the *Lp*.

**Figure 8:**
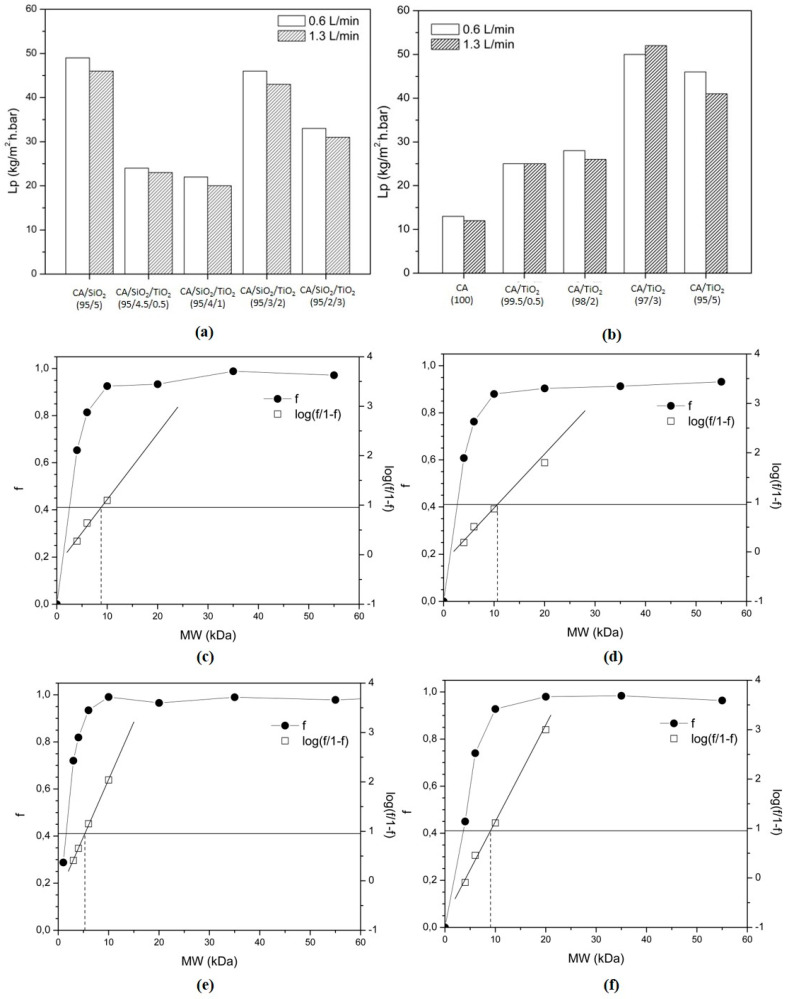
(**a**,**b**) Hydraulic permeability (*Lp*) for the membranes in series 1 (**a**) and 2 (**b**) determined at TMPs 0.5, 1.0, 1.5, 2.0, 2.5, and 3.0 bar and feed flow rate 0.6 L/min (white), and 1.3 L/min (hatched). (**c**–**f**) Rejection curves to PEGs of increasing MW and determination of the MWCO of the membranes in series 1 (**c**,**d**) and series 2 (**e**,**f**); (**c**) CA/SiO_2_ (95:5) membrane: *f* vs. MW curve (black circle) log(f/1 − f) (square), MWCO 9 kDa; (**d**) CA/SiO_2_/TiO_2_ (95:2:3) membrane: *f* vs. MW curve (black circle) log(f/1 − f) (square), MWCO 11 kDa; (**e**) CA (100) membrane: *f* vs. MW curve (black circle) log(f/1 − f) (square), MWCO 5 kDa; and (**f**) CA/TiO_2_ (95:5) membrane: *f* vs. MW curve (black circle) log(f/1 − f) (square), MWCO 9 kDa.

Hydraulic permeability coefficient (*Lp*) values for all hybrid membranes of series 1 and series 2 at 0.6 L/min (black) and 1.3 L/min (gray) are shown in Figure 8a,b, respectively. The *Lp* values were determined by the linear regressions of *Jpw* vs. ∆*P* for all membranes and the two flow rates. For all membranes in series 1 and series 2, an average value of the *Lp* was determined from the *Lp* values found for each flow rate.

For the membranes in series 1, the values of *Lp* were 47.5, 23.5, 21.0, 44.5, and 32 kg/m^2^.h.bar for the membranes containing 0, 0.5, 1, 2 and 3 wt% TiO_2_ (Figure 8a). The value for the first membrane of series 1, containing only CA and SiO_2_, is in the same value range found in our previous work regarding CA/SiO_2_ membranes [24]. When compared to the reference membrane in series 1, CA/SiO_2_ (95/5), the incorporation of TiO_2_ reflects a decrease in the hydraulic permeability of all hybrid CA/SiO_2_/TiO_2_ membranes. Another factor that influences the *Lp* is the thickness of the hybrid membranes’ skin dense layer (Figure 4b), where thicker dense layers offer higher resistance to water flux and which is in agreement with the results obtained from the permeation experiments, as the hybrid CA/SiO_2_/TiO_2_ (95/4.5/0.5) and CA/SiO_2_/TiO_2_ (95/4/1) membranes showed thicker skin dense layers (271.0 ± 89.0 and 213.0 ± 51.0 nm, respectively) than the reference CA/SiO_2_ (95/5) membrane (194.0 ± 18.0 nm), leading to a reduction of 50–60% in *Lp* values. In contrast, the skin layer thicknesses of the hybrid CA/SiO_2_/TiO_2_ (95/3/2) and CA/SiO_2_/TiO_2_ (95/2/3) membranes were smaller (142.0 ± 57.0 and 166.0 ± 58.0 nm, respectively) than that of the reference membrane and they still exhibited lower *Lp* values than the reference membrane. Even though no direct correlation between the TiO_2_ content and the *Lp* of the membranes in series 1 can be established, we can conclude that the introduction of TiO_2_ has an effect in the organization of the molecular structure of the hybrid material at the dense layer surface. The molecular organization of the final hybrid material possesses open spaces (pores at the nano- and sub-nanoscale not detected by SEM) at the top dense surface of the membranes, which allow the water molecules to flow through the membranes. Taking this into account, the membranes containing TiO_2_ seem to have a tighter structure, which hinders the passage of water and other solutes, resulting in a lower *Lp*. For the membranes in series 2, the values of *Lp* were 12.5, 25.0, 27.0, 51.0, and 43.5 kg/m^2^.h.bar for the membranes containing 0, 0.5, 2, 3 and 5 wt% TiO_2_ (Figure 8b). When compared to the series 2 reference membrane, CA (100), the incorporation of TiO_2_ increased the hydraulic permeability of all hybrid CA/TiO_2_ membranes by a factor of at least 2. All hybrid CA/TiO_2_ membranes in series 2 have similar dense layer thicknesses (≈180 nm), which are approximately 1.8 smaller than the dense layer thickness of the pristine CA membrane (reference membrane for series 2). It is, therefore, expected that the *Lp* values for the hybrid membranes should be higher than the value for the pristine CA membrane and this is in accordance with the results. The interesting fact is that the *Lp* continues to increase as the TiO_2_ content increases in the hybrid CA/TiO_2_ membranes, even though the dense layer thickness remains essentially the same. This suggests that, as the TiO_2_ content increases the molecular structure of the hybrid CA/TiO_2_ membrane, the material is modified, promoting larger open spaces and a looser overall structure which facilitates the flux of water molecules, resulting in a higher *Lp*.

In summary, the introduction of titania in the membranes in series 1, CA/SiO_2_/TiO_2_ hybrid membranes, did not improve the water permeation of the CA/SiO_2_ (95/5) reference membrane. In contrast, the introduction of titania in the membranes in series 2, CA/TiO_2_ membranes, improved the water permeation of the pristine CA membrane and the incremental increase in TiO_2_ further increased the *Lp*, with both hybrid CA/TiO_2_ (97/3) and CA/TiO_2_ (95/5) membranes being the ones with the highest *Lp* values.

For the series 1 and series 2 membranes, the MWCO was determined for the reference membranes (non-TiO_2_ containing membranes), CA/SiO_2_ (95:5) (Figure 8c) and CA (100) (Figure 8e), and for the membranes containing the highest TiO_2_ content, CA/SiO_2_/TiO_2_ (95:2:3) (Figure (8d) and (Figure 8d) CA/TiO_2_ (95:5). For the membranes in series 1, the MWCO values of CA/SiO_2_ (95:5) and CA/SiO_2_/TiO_2_ (95:2:3) were 9 and 11 kDa, respectively. As was seen for the values of the *Lp*, the introduction of TiO_2_ does not seem to have a significant effect on the structures of the hybrid material which composes the dense layer of the membranes in series 1. For the membranes in series 1 containing 0 wt% titania and the highest content of titania (3 wt%), the rejection to macromolecules with MW 10 kDa is higher than 90%.

For the membranes in series 2, the MWCO value of the pristine CA membrane, CA (100), was 5 kDa and, for the membrane containing the highest TiO_2_ content, CA/TiO_2_ (95:5), 9 kDa. As seen from the results obtained for the *Lp* values of the series 2 membranes, the introduction of TiO_2_ does influence the final structure of the hybrid material which composes the dense layer of the CA/TiO_2_ membranes when compared to the pristine CA membrane. Once again, the introduction of TiO_2_ seems to increase the size of the open spaces at the dense layer surface, enabling the passing/movement of larger molecules through the membranes containing titania when compared to the pristine CA membrane.

In series 2, the MWCO measured for the pure CA membrane was 5 kDa and, for the CA/TiO_2_ (95/5) hybrid membrane, 9 kDa. In this case, the introduction of 5 wt% TiO_2_ also opened the membrane pores in the skin dense layer and let larger molecules cross. These results are in agreement with the *Lp* values determined for series 2 membranes. The pristine CA membrane showed a lower pure water permeate flux than the CA/TiO_2_ (95/5) hybrid membrane, which already indicates that the membrane pores opened with TiO_2_ introduction. Nevertheless, both series of membranes are classified as ultrafiltration membranes, since their size exclusion ranges from 0.5 to 100 kDa [47].

## 4. Conclusions

Two series of novel integral asymmetric monophasic hybrid membranes, CA/SiO_2_/TiO_2_ (series 1) and CA/TiO_2_ (series 2), were developed by the coupling of sol-gel technology and a modified version of the phase inversion technique, a method pioneered by the authors [24]. For the series 1 membranes, the organic content (CA) was kept constant and equal to 95 wt% in all membranes. The reference membrane, CA/SiO_2_ (95:5), contained 5 wt% SiO_2_ and, in the other four hybrid CA/SiO_2_/TiO_2_ membranes, the inorganic content was a combination of SiO_2_ and TiO_2_, with TiO_2_ steadily replacing silica (from 0 to 3 wt%). For the membranes in series 2, the organic content (CA) was kept constant and equal to 95 wt%. The inorganic phase, composed solely of TiO_2_, was increased from 0 wt%, in the reference CA (100) membrane to 5 wt%. The membranes were characterized in terms of surface and cross-section structure by SEM, chemical composition by ATR-FTIR, and ICP-OES, and the permeation performance was evaluated in terms of *Lp* and MWCO.

SEM micrographs confirmed the integral asymmetric structure of all membranes in series 1 and series 2 and indicated that there is no direct correlation between the introduction of TiO_2_ and the total membranes’ thickness. In terms of the dense layer thickness, the introduction of TiO_2_ seems to promote thinner dense layers for the hybrid CA/TiO_2_ membranes (series 2) when compared to the reference CA (100) membrane, while no correlation was found for the membranes in series 1.

ICP-OES revealed that the amount of Ti present in the samples of all membranes in series 1 and series 2 was equal to the amount introduced in the casting solution in the form of TiPOT. Furthermore, ICP-OES confirmed that no Ti was leached from the membranes into the permeate or feed solution during typical filtration experiments performed at 2 bar for 3 h.

ATR-FTIR proved the existence of a monophasic hybrid network composed of cellulose–acetate and silica and/or titania covalently connected by Si–O–C and Ti–O–C covalent bonds. Furthermore, in the membranes from series 2, CA/TiO_2_ membranes, results show that TiO_2_ preferably bonds to CA by nucleophilic substitution of the hydroxyl group rather than the carbonyl group.

The results obtained from ICP-OES and ATR-FTIR lead us to conclude that, in both membrane series, Ti is directly (series 2) or indirectly (series 1) covalently bound to the polymer matrix, forming two novel monophasic hybrid organic-inorganic materials: CA/SiO_2_/TiO_2,_ and CA/TiO_2_. In the CA/SiO_2_/TiO_2_ membranes, Ti is covalently linked to the silica groups which, in turn, are covalently linked to the CA, while, in the CA/TiO_2_ membranes, Ti is directly linked to CA.

Permeation experiments revealed that the introduction of titania in the membranes in series 1 did not improve the water permeation when compared to the reference membrane, CA/SiO_2_ (95/5). In contrast, the introduction of titania in the membranes in series 2 increased the water permeation by a factor of at least 2 when compared to the reference membrane, CA (100), and the incremental increase in TiO_2_ in the CA/TiO_2_ membranes further increased the *Lp*, with the hybrid membranes containing TiO_2_, CA/TiO_2_ (97/3) and CA/TiO_2_ (95/5) being the ones with the highest *Lp* values. Following the same trend, the MWCO of the membranes in series 1 was unaffected by the introduction of TiO_2_, while, for the membranes in series 2, the introduction of 3 wt% TiO_2_ increased the MWCO from 5 kDa for the pristine CA membrane, CA (100), to 10 kDa for the CA/SiO_2_/TiO_2_ (95:2:3) membrane.

## Figures and Tables

**Table 1:** Membrane acronyms and casting solution composition (series 1 and series 2).

**Acronyms/Compounds**	**Series 1**				
	**CA/SiO_2_** **(95/5)**	**CA/SiO_2_/TiO_2_ (95/4.5/0.5)**	**CA/SiO_2_/TiO_2_ (95/4/1)**	**CA/SiO_2_/TiO_2_ (95/3/2)**	**CA/SiO_2_/TiO_2_** **(95/2/3)**
CA	16.40	16.40	16.40	16.40	16.40
Formamide	29.00	29.00	29.00	29.00	29.00
Acetone	51.10	51.10	51.10	51.10	51.10
TEOS	3.00	2.70	2.40	2.10	0.93
TiPOT	0.00	0.32	0.64	0.96	2.07
DI	0.50	0.5	0.5	0.5	0.5
HNO_3_	3 drops (pH 1.5–2)				
**Acronyms/Compounds**	**Series 2**				
	**CA** **(100)**	**CA/TiO_2_ (99.5/0.5)**	**CA/TiO_2_** **(98/2)**	**CA/TiO_2_** **(97/3)**	**CA/TiO_2_** **(95/5)**
CA	17.00	16.40	16.40	16.40	16.40
Formamide	30.00	29.00	29.00	29.00	29.00
Acetone	53.00	51.10	51.10	51.10	51.10
TEOS	-	-	-	-	-
TiPOT	0.00	0.32	0.96	2.07	3.00
DI	0.00	0.5	0.5	0.5	0.5
HNO_3_	3 drops (pH 1.5–2)				
**Casting Conditions**					
Temperature (°C): 19–23					
Solvent evaporation time (s): 30					
Coagulation medium: Deionized water at 0 °C during 1–2 h					

NOTE 1: reference membranes in blue column. NOTE 2: the membranes’ acronyms reflect the final membranes’ composition in terms of wt% and not the casting solution compositions.

**Table 2:** Ti present in casting solutions (Ti_theoretical_) and in the membranes (Ti_experimental_) from series 1 and 2.

**Series 1**	**Ti_(theoretical)_ (mg)**	**Ti_(exp)_ (mg)**
CA/SiO_2_ (95/5)	0.00 ± 0.01	0.00 ± 0.10
CA/SiO_2_/TiO_2_ (95/4.5/0.5)	0.20 ± 0.01	0.20 ± 0.10
CA/SiO_2_/TiO_2_ (95/4/1)	0.44 ± 0.01	0.40 ± 0.10
CA/SiO_2_/TiO_2_ (95/3/2)	0.62 ± 0.01	0.70 ± 0.10
CA/SiO_2_/TiO_2_ (95/2/3)	1.28 ± 0.01	1.20 ± 0.20
**Series 2**	**Ti_(theoretical)_ (mg)**	**Ti_(exp)_ (mg)**
CA (100)	0.00 ± 0.01	0.00 ± 0.10
CA/TiO_2_ (99.5/0.5)	0.31 ± 0.01	0.50 ± 0.10
CA/TiO_2_ (98/2)	0.76 ± 0.01	1.30 ± 0.30
CA/TiO_2_ (97/3)	1.66 ± 0.01	1.30 ± 0.50
CA/TiO_2_ (95/5)	2.39 ± 0.01	2.00 ± 0.20

**Table 3:** Assignments of the ATR-FTIR spectra of the CA/SiO_2_/TiO_2_ (series 1) membranes.

Wavenumber/cm^−1^					Assignment
CA/SiO_2_ (95/5)	CA/SiO_2_/TiO_2_ (95/4.5/0.5)	CA/SiO_2_/TiO_2_ (95/4/1)	CA/SiO_2_/TiO_2_ (95/3.5/1.5)	CA/SiO_2_/TiO_2_ (95/2/3)	
3487 ^br^	3490	3491	3489	3490	νOH (H bonded)
1741 ^S^	1742	1743	1736	1747	νC=O
1637 ^w^	1637	1637	1637	1638	δHOH (H_2_O)
1433 ^w^	1432	1432	1431	1432	δCH_2_
1369 ^m^	1369	1369	1369	1369	δ_s_CH_3_
1232 ^VS^	1232	1232	1233	1232	ν_as_COC (acetate)
1161 ^w^	1161	1161	1161	1161	ν_as_COC (glycosidic)
1122 ^w,sh^	1122	1122	1122	1122	ν_as_COC (glycosidic)
1065 ^w^	1065	1065	1065	1065	ν_as_COC (glycosidic), ν(Si–O–Si)
1045 ^VS^	1045	1045	1045	1045	ν_s_COC (acetate)
903 ^w^	904	903	903	904	ρCH_3_

VS—very strong; S—strong; m—medium; w—weak; vw—very weak; sh—shoulder; br—broad.

**Table 4:** Areas and wavenumbers of the carbonyl group located in the 1690–1800 cm^−1^ region for the series 1 membranes.

Components	CA/SiO_2_ (95/5)		CA/SiO_2_/TiO_2_ (95/4.5/0.5)		CA/SiO_2_/TiO_2_ (95/4/1)		CA/SiO_2_/TiO_2_ (95/3.5/1.5)		CA/SiO_2_/TiO_2_ (95/2/3)	
	$\tilde{\nu }$/cm^−1^	A	$\tilde{\nu }$/cm^−1^	A	$\tilde{\nu }$/cm^−1^	A	$\tilde{\nu }$/cm^−1^	A	$\tilde{\nu }$/cm^−1^	A
	1741	27.67	1742	23.87	1743	23.30	1746	22.80	1747	23.40

**Table 5:** Summary of the spectral deconvolution in the 1000–1110 cm^−1^ region in series 1 (CA/SiO_2_/TiO_2_).

Components	CA/SiO_2_ (95/5)		CA/SiO_2_/TiO_2_ (95/4.5/0.5)		CA/SiO_2_/TiO_2_ (95/4/1)		CA/SiO_2_/TiO_2_ (95/3.5/1.5)		CA/SiO_2_/TiO_2_ (95/2/3)	
	$\tilde{\nu }$/cm^−1^	A (%)	$\tilde{\nu }$/cm^−1^	A (%)	$\tilde{\nu }$/cm^−1^	A (%)	$\tilde{\nu }$/cm^−1^	A (%)	$\tilde{\nu }$/cm^−1^	A (%)
	1039	51	1040	53	1040	57	1039	63	1039	64
	1066	49	1067	47	1068	43	1068	37	1067	36

**Table 6:** Assignments of the ATR-FTIR spectra of the CA/TiO_2_ (series 2) membranes.

Wavenumber/cm^−1^					Assignment
CA(100)	CA/TiO_2_ (99.5/0.5)	CA/TiO_2_ (98/2)	CA/TiO_2_ (97/3)	CA/TiO_2_ (95/5)	
3490 ^br^	3488	3494	3500	3500	νOH (H bonded)
1739 ^S^	1744	1745	1749	1749	νC=O
1635 ^w^	1639	1636	1639	1639	δHOH (H_2_O)
1435 ^w^	1437	1430	1433	1433	δCH_2_
1368 ^m^	1379	1369	1379	1373	δ_s_CH_3_
1233 ^VS^	1234	1234	1234	1228	ν_as_COC (acetate)
1165 ^w^	1163	1163	1163	1163	ν_as_COC (glycosidic)
1037 ^VS^	1043	1043	1043	1043	ν_s_COC (acetate)
898 ^w^	903	903	897	903	ρCH_3_

VS—very strong; S—strong; m—medium; w—weak; vw—very weak; sh—shoulder; br—broad.

**Table 7:** Areas and wavenumbers of the carbonyl group located in the 1690–1800 cm^−1^ region for the CA/TiO_2_ membranes.

Components	CA (100)		CA/TiO_2_ (99.5/0.5)		CA/TiO_2_ (98/2)		CA/TiO_2_ (97/3)		CA/TiO_2_ (95/5)	
	$\tilde{\nu }$/cm^−1^	A	$\tilde{\nu }$/cm^−1^	A	$\tilde{\nu }$/cm^−1^	A	$\tilde{\nu }$/cm^−1^	A	$\tilde{\nu }$/cm^−1^	A
	1739	18.69	1744	22.35	1745	22.41	1749	22.80	1749	23.09

**Figure A1:**
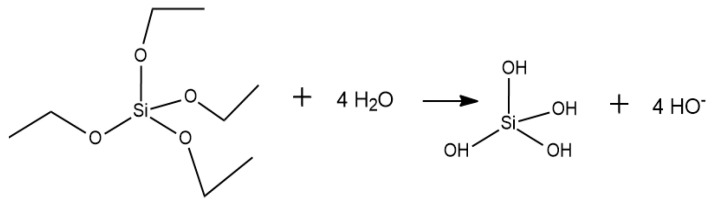
Hydrolysis of TEOS.

**Figure A2:**
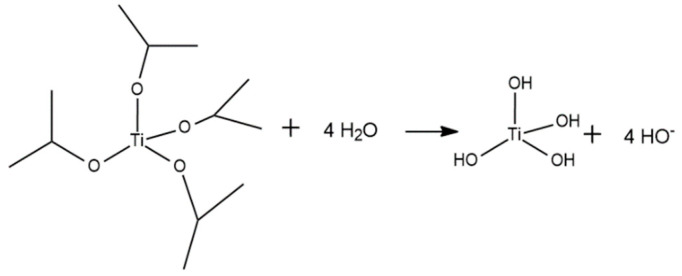
Hydrolysis of TiPOT.

**Figure A3:**
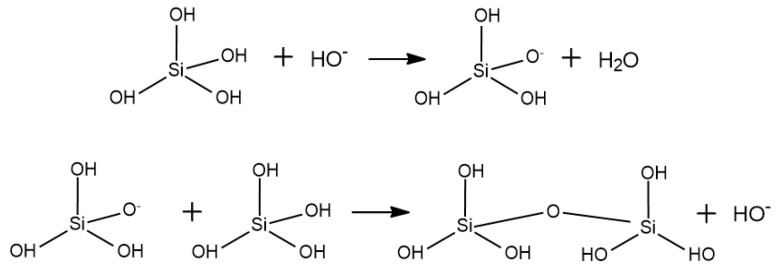
Two step homo-condensation: between orthosilic Si(OH)_4_ molecules.

**Figure A4:**
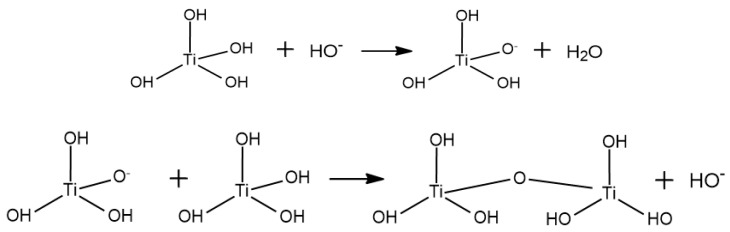
Two step homo-condensation: between Ti(OH)_4_ molecules.

**Figure A5:**
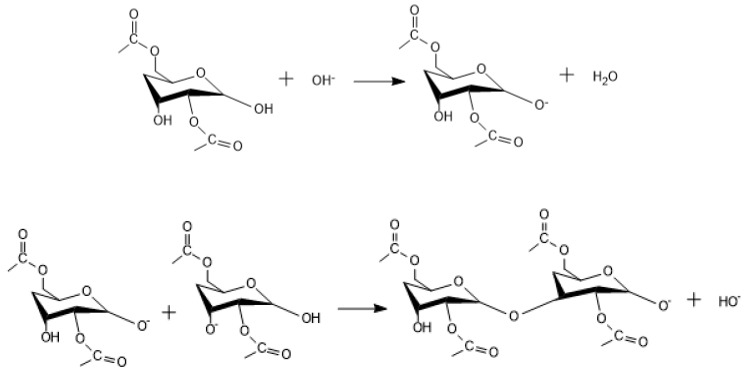
Two step homo-condensation: between CA–CA polymer molecules.

**Figure A6:**
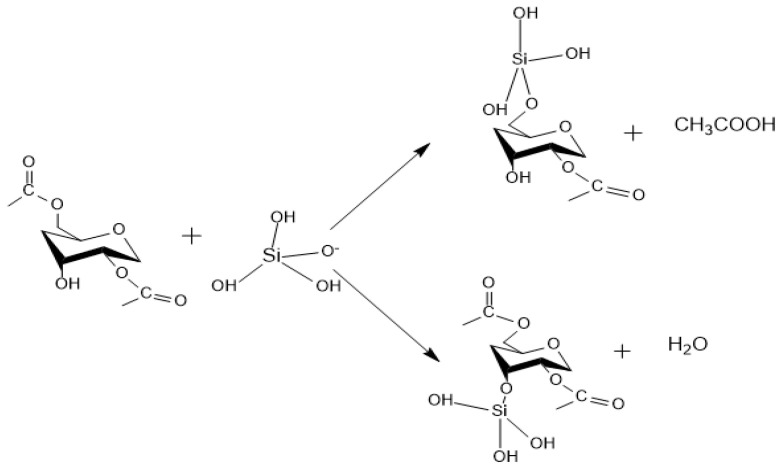
Hetero-condensation between Si(OH)_4_ and CA polymer molecules.

**Figure A7:**
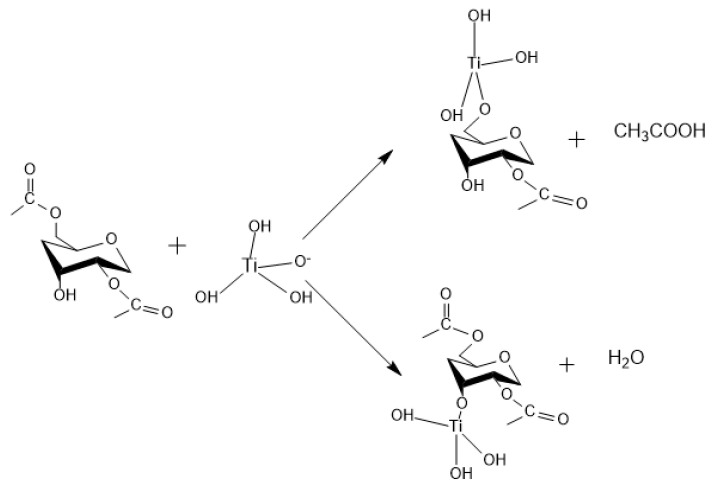
Hetero-condensation between Ti(OH)_4_ and CA polymer molecules.

**Figure A8:**
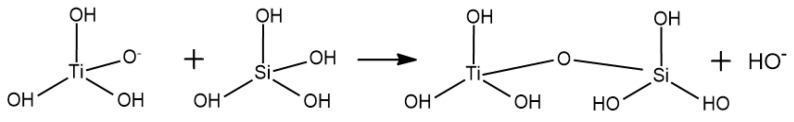
Hetero-condensation between Si(OH)_4_ and Ti(OH)_4_ molecules.

**Figure A9:**
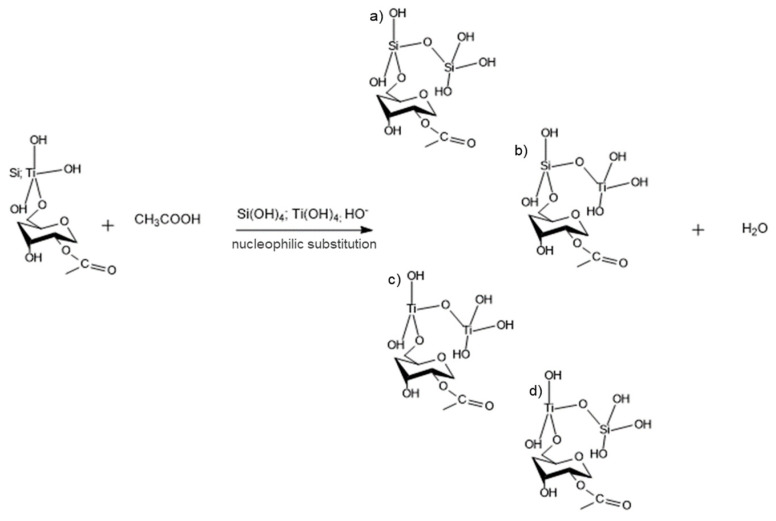
Further condensation reactions between Si(OH)_4_ and/or Ti(OH)_4_ molecules with other inorganic molecules already bonded to the CA polymer.

**Figure A10:**
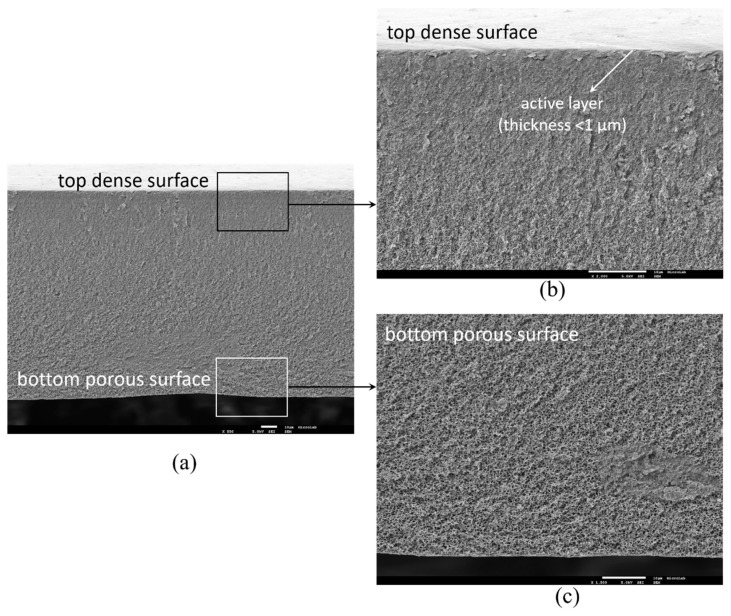
FEG-SEM images of the cross section structure of the CA/SiO_2_/TiO_2_ (95:4:1) membranes: (**a**) image of the total cross section structure (550×), (**b**) image of a region near the top dense surface where the active layer thickness can be seen (2000×), and (**c**) image of a region near the bottom porous surface (1500×).

## Appendix A. Acid Catalyzed Sol-Gel Hydrolysis and Condensation Reactions

The sol-gel process develops during the homogenization of the casting solution (24 h period) under acid catalysis and is responsible for the covalent binding of SiO_2_ and TiO_2_ to the CA network. The sol-gel process consists in hydrolysis and condensation reactions which take place according to the following order:

Immediately after the addition of catalysts (HNO_3_), TEOS starts hydrolyzing (at a very slow rate). As soon as TIPOT is added to the casting solution, its hydrolysis occurs (at a very high rate, almost instantly). After complete hydrolysis of both SiO_2_ and TiO_2_ precursors (TEOS and TIPOT, respectively), homo- and hetero-condensation takes place.

Homo-condensation reactions include: homo-condensation between orthosilic Si(OH)_4_ molecules, homo-condensation between Ti(OH)_4_ molecules, and homo-condensation between CA–CA polymer. Hetero-condensation reactions include: hetero-condensation between Si(OH)_4_ and CA polymer, hetero-condensation between Ti(OH)_4_ and CA polymer, and hetero-condensation between between Si(OH)_4_ and Ti(OH)_4_.

As the condensation reactions take place, other Si(OH)_4_ or Ti(OH)_4_ molecules may condensate with other inorganic molecules already bonded to the CA polymer.

## Appendix B

Calculation base: 100 g casting solution
M (TEOS) = 208.33 gmol^−1^ M(SiO_2_) = 60.0843 gmol^−1^
M(TiPOT) = 284.22 gmol^−1^ M(TiO_2_) = 79.8658 gmol^−1^

**Table A1:** Membrane Acronyms and Casting Solution Composition (series 1 and series 2).

**ACRONYM**	**Casting Solution Composition (wt%)**					
**CA/SiO_2_/TiO_2_**	**TEOS**	**SiO_2_**	**TiPOT**	**TiO_2_**	**CA**	**(CA + SiO_2_ + TiO_2_)**
95/5/0	3	0.865228	0	0	16.4	17.26523
95/4.5/0.5	2.7	0.778705	0.32	0.08992	16.4	17.26863
95/4/1	2.4	0.692182	0.64	0.17984	16.4	17.27202
95/3/2	2.1	0.605659	0.96	0.269761	16.4	17.27542
95/2/3	0.93	0.268221	2.07	0.581671	16.4	17.24989
**ACRONYM**	**Casting Solution Composition (wt%)**					
**CA/TiO_2_**			**TiPOT**	**TiO_2_**	**CA**	**(CA + TiO_2_)**
100			0	0	17	17
99.5/0.5			0.32	0.08992	16.4	16.48992
98/2			0.96	0.269761	16.4	16.66976
97/3			2.07	0.581671	16.4	16.98167
95/5			3	0.843002	16.4	17.243

## Appendix C

**Table A2:** Membrane drying process: stages, solvents, and immersion time.

Stage	Solution	Time (h)
1	H_2_Od	24
2	Aqueous solution of Isopropanol (25% V/V)	
3	Aqueous solution of Isopropanol (50% V/V)	
4	Aqueous solution of Isopropanol (75% V/V)	
5	Solution of Isopropanol (100% V/V)	
6	Solution of Isopropanol (75% V/V) + n–Hexane (25% V/V)	
7	Solution of Isopropanol (50% V/V) + n–Hexane (50% V/V)	
8	Solution of Isopropanol (25% V/V) + n–Hexane (75% V/V)	
9	Solution of n–Hexane (100% V/V)	
10	Desiccator	

## Appendix D. Estimation of the Dense Layer Thickness from FEG-SEM cross Section Images and ImageJ Software

The FEG-SEM figure illustrates the cross section of the CA/SiO_2_/TiO_2_ (95:4:1) membrane (a) where the regions near the top dense (b) and near the bottom porous surface (c) are shown at higher amplifications. In image (b), the active layer, characterized by a very thin dense layer on top of a porous substructure, is visible at a large scale and its thickness can be measured using ImageJ software. It is also possible to see that the pores throughout the cross section (from top to bottom) become larger in the direction from the active layer to the bottom porous surface. Image (c) shows an amplified region near the bottom porous surface, where pores are larger than the ones neighboring the active layer. Images with these amplifications were taken for all membranes (series 1 and series 2) in order to calculate the active layer thickness. The figure shows that the total thickness of the membranes in series 1 was calculated from the images in the third column using ImageJ software.

## Appendix E. Titanium Leached from the Membranes

**Table A3:** Ti concentration ([Ti]) (mg/L) of the initial H_2_Od sample and of the final H_2_Od samples collected from the permeate and concentrate cells in the permeation assay. CA/SiO_2_/TiO_2_ (95/2/3) and CA/TiO_2_ (95/5) membranes were tested.

**Series 1**	**H_2_Od Sample**	**[Ti] (mg/L)**
CA/SiO_2_/TiO_2_ (95/2/3)	Initial	<0.010 ± 0.000
	Final permeate	<0.010 ± 0.000
	Final concentrate	<0.010 ± 0.000
**Series 2**	**H_2_Od Sample**	**[Ti] (mg/L)**
CA/TiO_2_ (95/5)	Initial	<0.010 ± 0.000
	Final permeate	<0.010 ± 0.000
	Final concentrate	<0.010 ± 0.000
